# Effects of a 6-week horizontal speed deceleration training on measures of physical fitness in youth tennis players

**DOI:** 10.5114/biolsport.2026.159558

**Published:** 2026-04-20

**Authors:** Yassine Negra, Senda Sammoud, Raja Bouguezzi, Jason Moran, Rodrigo Ramirez-Campillo, Younés Hachana, Helmi Chaabene

**Affiliations:** 1 Research Laboratory (LR23JS01) «Sport Performance, Health & Society»; 2 Higher Institute of Sport and Physical Education of Ksar Saïd, University of “La Manouba”, Tunisia, 2037; 3 Université de Jendouba, Institut Supérieur de Sport et de l’Education Physique du Kef, 7100, Le Kef, Tunisie; 4 School of Sport, Rehabilitation, and Exercise Sciences, University of Essex, Colchester, United Kingdom; 5 Exercise and Rehabilitation Sciences Institute. Faculty of Rehabilitation Sciences. Universidad Andres Bello. Santiago 7591538, Chile; 6 Department of Physical Activity Sciences. Universidad de Los Lagos. Osorno, Chile; 7 University Hospital Magdeburg, Department of Cardiology and Angiology, Otto-von-Guericke University, Magdeburg, 39120 Magdeburg, Germany

**Keywords:** Human physical conditioning, Eccentric training, Eccentric strength, Racquet sports, Pubertal athletes

## Abstract

Horizontal-speed-deceleration-training (HSDT) has recently emerged as a promising method that specifically targets the eccentric actions of the knee-extensors. This study aimed to compare the effects of replacing a portion of traditional in-season tennis training with HSDT compared to standard training alone on the physical fitness of youth tennis players. Tennis players (n = 23) from two regional tennis teams were assigned to the HSDT-group (n = 14 [8 males, 6 females]; age = 13.24 ± 0.60 years) or to the control-group (n = 9 [6 males, 3 females]; age = 13.08 ± 0.56 years). Before-and- after six weeks of training, participants were assessed for linear 20-m sprint-speed, change-of-direction (COD) speed (505 test), agility (Y-shaped-agility test), jump height/distance (countermovement-jump [CMJ] height; 20-cm drop-jump height; standing-longjump [SLJ] distance), reactive-strength-index (RSI), and repeated-sprint-ability (RSA). ANOVA revealed significant Group-by-Time interactions, indicating greater improvements in the HSDT-group compared to the control-group in 20 m sprint-speed, COD-speed, agility, CMJ-height, SLJ-distance, 20 cm drop-jump height, and RSI (ES = 0.87–2.14; p < 0.05), whereas no significant interaction was observed for RSA outcomes. The HSDT group improved 20-m linear sprint-time (ES = 1.19 [moderate], p < 0.001), COD-speed (ES = 1.19 [moderate], p < 0.001), agility (ES = 2.14 [large], p < 0.01), CMJ-height (ES = 1.42 [large], p < 0.01), SLJ-distance (ES = 1.05 [moderate], p < 0.05), 20-cm drop-jump height (ES = 0.87 [moderate]; p < 0.05), and RSI (ES = 1.71 [large], p < 0.01). However, no significant pre-to-post changes were observed in the group (ES = 0.09 to 0.13; p > 0.05). Replacing part of regular in-season tennis training with HSDT appears to be highly effective in improving multiple physical fitness aspects in youth tennis players. Regular tennis training alone seems insufficient to elicit meaningful gains in these key physical qualities.

## INTRODUCTION

Success in tennis depends on the integration of multiple physical attributes, including muscular strength, power, speed, agility, and endurance [1–3]. Indeed, elite tennis players have consistently displayed higher performance in distinct physical abilities when compared to amateur players [3]. The high level of physical fitness in elite players plays a crucial role in helping them accommodate the intense demands of training and competition in modern tennis. Accordingly, the early development of key fitness performance components of tennis is paramount to prepare youth players for the heightened training and competition requirements in contemporary tennis.

Although natural growth contributes to improvements in physical capacities during adolescence [4] targeted training interventions are essential to optimize athletic development [5, 6]. Therefore, it is imperative to develop and implement well-structured training interventions that address the specific fitness demands of tennis players in competition to increase the chance of achieving sporting success in their future careers.

Several training interventions, such as plyometric training [2, 7] and resisted or unresisted sprint training [8, 9] can generate beneficial effects on measures of physical fitness in youth tennis players. More specifically, over the last decade, eccentric training has gained recognition for its significant benefits in enhancing physical fitness across various sports [10–12]. In this context, Bright et al. [12] conducted a systematic review and indicated that there is strong evidence supporting the systematic integration of eccentric resistance training to improve a wide range of measures of physical fitness in youth athletes. While eccentric-based methods such as the Nordic Hamstring Exercise and flywheel training have demonstrated significant benefits, their application in tennis remains limited, particularly among youth players. Several (accentuated) eccentric training methods (e.g., Nordic Hamstring Exercise, flywheel inertial training) have been used as effective tools for improving measures of physical fitness in both male and female athletes [12]. Therefore, the present study aims to address this gap by investigating the effects of eccentric-based training in youth tennis players. To the authors’ knowledge, eccentric training methods are rarely applied to tennis players, especially with youth players. Additionally, due to the limited choice of (accentuated) eccentric exercises currently available, the need for a more varied selection of effective exercises is increasing. HSDT has been proposed as a practical method to target the eccentric actions of the knee extensors, with previous evidence demonstrating improvements in change of direction and jumping ability [13, 14] studied the effect of HSDT on measures of physical fitness in youth male handball players aged 15 years. They reported that an in-season HSDT performed alongside regular handball-specific training resulted in large [ES = 1.07 to 1.70] beneficial effects on measures of COD speed, jumping ability, and RSA performance compared to non-significant effects in the active control group.

In tennis, deceleration actions are crucial for players to effectively control their movements and execute shots with precision [15]. This involves stopping their momentum efficiently to set up for forehands, backhands, volleys, or smashes. Therefore, a proper deceleration ensures that tennis players are balanced and stable when striking the ball [15]. Likewise, tennis players frequently change direction on the court, requiring rapid deceleration to stop their movement before accelerating in a new direction. This ability to control deceleration helps in maintaining agility, reacting to opponents’ shots, and covering the court effectively [15].

In light of these facts, youth tennis coaches are encouraged to emphasize sport-specific training, such as that based upon HSDT, alongside other aspects of fitness to optimize performance. Despite the importance of deceleration ability in tennis, no studies have directly examined the effects of HSDT on physical fitness parameters in youth tennis players. Notably, to the best of the authors’ knowledge, there are only two studies [14, 16] that examined the effects of HSDT on measures of physical fitness in young athletes, but not in tennis. This study aimed to examine the effects of a six-week HSDT intervention on various measures of physical fitness in youth tennis players, hypothesizing that HSDT would elicit greater improvements than standard tennis training alone [14].

## MATERIALS AND METHODS

### Participants

With reference to the study of Negra et al. [14] an a priori power analysis, with a type I error rate of 0.05 and 80% statistical power, was computed. The analysis indicated that a total of 20 participants would be sufficient to observe a significant interaction effect (effect size Cohen’s *d* = 1.10 for the RSA_best_). To address potential participant attrition, a total of 23 pubertal tennis players were assigned to either the HSDT group (n = 14; 8 males and 6 females) or the active CG (n = 9; 6 males and 3 females). All participants were experienced tennis players (mean training experience: 8.0 ± 1.1 years) and were free of injury for at least six months prior to the study. Table 1 presents the anthropometric data for both groups. The maturity offset method was used to assess the biological maturity of participants [17]. The following valid prediction equations were applied:

For boys: maturity offset = −7.999994 + (0.0036124*age*height).

For girls: maturity offset = −7.709133 + (0.0042232 *age*height).

**TABLE 1:** Anthropometric characteristics of the included participants

	HSDT group (n= 14)	CG (n= 9)
Age (years)	13.24 ± 0.60	13.08 ± 0.56
Body height (cm)	166.86 ± 6.80	165.67 ± 10.99
Body mass (kg)	54.57 ± 10.28	55.67 ± 10.21
Maturity offset (years)*	0.62 ± 0.96	-0.17 ± 0.66
APHV	13.26 ± 0.38	13.25 ± 0.54

Notes: Data are presented as means and standard deviations; HSDT = horizontal speed deceleration training; CG = control group;

* : as years from peak height velocity. APHV = age at peak height velocity.

All the experimental procedures and the associated potential risks were fully explained and written informed consent (parents/legal guardians) and assent (participants) were obtained before the commencement of the study. All procedures were approved by the local Institutional Review Committee of the Higher Institute of Sport and Physical Education of Ksar Said (LR23JS25), and conducted in accordance with the latest version of the Declaration of Helsinki.

### Experimental design

We examined whether 6 weeks of biweekly in-season HSDT would enhance various measures of physical fitness in youth tennis players relative to their peers who maintained their customary in-season training regimen. Two regional youth tennis teams were assigned to the HSDT or control group. Both groups completed five weekly training sessions, with the HSDT group replacing 25 minutes of low-intensity tennis drills with HSDT on two occasions per week. After the HSDT portion of the session, players completed the remainder of their regular tennis-specific training. Two weeks before baseline testing, two familiarization sessions were performed. The following tests were performed before and after the training program: 20-m linear sprinting speed, 505 COD speed test, Y-shaped agility test, countermovement jump (CMJ), 20-cm drop jump (DJ-20), standing long jump (SLJ), RSI, and RSA. All tests were scheduled at least 48 hours after the last training session or match, at the same time of day (18:00–19:30).

### Horizontal speed deceleration training program

The HSDT program was conducted during the second half of the in-season period (April-May, 2024). Prior to every HSDT session, a standardized 8–12-min warm-up was completed, including low-intensity running, coordination exercises, dynamic movements (i.e., lunges, skips), sprints, and dynamic stretching for the lower-limb muscles. At the beginning of each training week, the first HSDT session was performed at least 48 hours after the last tennis match that was scheduled on the weekend. The second HSDT session was completed 72-h after the first session (i.e., Tuesday and Friday). The HSDT drills were performed at the beginning of the tennis training session. The HSDT protocol is detailed in Table 2. The total running distance (acceleration + deceleration) per week gradually increased from 300-m during the first week to 500-m during the last week of training. Players were instructed to (i) focus on taking shorter, controlled steps, (ii) maintain an upright posture with a slight forward lean in the deceleration phase, and (iii) keep the center of gravity low and the knees slightly bent to absorb the impact. Each HSDT session included 10-m acceleration distance followed by 5-m deceleration distance. Participants were instructed to exert maximal acceleration effort during the 10-m distance and begin decelerating upon reaching the 10-m finish line. Subsequently, they were instructed to fully decelerate and come to a complete stop within a 3 m distance.

**TABLE 2:** Horizontal speed deceleration training program

Week		Acceleration distance (m)	Deceleration distance (m)	Repetitions	Rest between repetitions (sec)	Rest between sets (min)
1	Set 1	10	3	8	90	4
	Set 2	10	3	8	90	

2	Set 1	10	3	10	90	4
	Set 2	10	3	10	90	

3	Set 1	10	3	12	90	4
	Set 2	10	3	12	90	

4	Set 1	10	3	10	90	4
	Set 2	10	3	10	90	

5	Set 1	10	3	12	90	4
	Set 2	10	3	12	90	

6	Set 1	10	3	10	90	4
	Set 2	10	3	10	90	

### Linear sprint speed time

Twenty-meter linear sprint performance was assessed using a single-beam electronic timing system (wittygate, Microgate, SRL, Bolzano, Italy). Participants started in a standing split stance position with their lead foot 0.3 m behind the first infrared photoelectric gate, which was placed 0.75 m above the ground to ensure that it captured trunk movement and avoided false signals through limb motion. In total, two single-beam photoelectric gates were used. No rocking or false steps were permitted before starting. The between-trial recovery time was 3 min. The best performance out of two trials was used for further analysis. The between-trial intraclass correlation coefficient (ICC) was 0.90.

### The 505 change of direction

The 505 COD speed test was administered per the protocol previously outlined by Negra et al. [14] using an electronic timing system (wittygate, Microgate, SRL, Bolzano, Italy). Players assumed a standing position 10 m from the start line, ran as quickly as possible through the start/finish line, pivoted 180° at the 15-m line indicated by a cone marker, and returned as fast as possible through the start/finish line. To ensure proper execution of the test, a researcher was positioned at the turning line and if the participant changed direction before reaching the turning point, the trial was disregarded and reattempted after 3 min recovery period. A between-trial rest period of three minutes was provided. The best performance out of two trials was used for further analysis. The between-trial ICC was 0.87.

### Y-agility shaped test

The Y-shaped agility test was administered using the protocol as previously outlined by Lockie et al. [16]. The Witty light-based timing system was used to record the time and set the reactive conditions. The width of the gates was 1.5 m with a height of 1.2 m. The participants began 0.3 m behind the start line and ran maximally in a 5 m straight sprint. Then they performed the change in direction task as quickly as possible with a 45_ cut to the left or to the right side followed by a 5 m-long sprint to the finish gates. As a stimulus, the green arrow was used to dictate the direction. It appeared with a delay of approximately 40–45 ms after passing the starting gate. Two trials were performed with a 90-second rest interval between them, and the best time was used for further analysis.

### Countermovement jump

During the CMJ, participants started from a standing position and performed a fast downward movement by flexing the knees and hips before rapidly extending the legs and performing a maximal vertical jump. During the test, participants were instructed to maintain their arms akimbo. Jump height was recorded using an optoelectric system (Optojump next, Microgate, SRL, Bolzano, Italy). A 90-second rest interval was provided between trials. The best out of three trials was retained for further analysis. The betweentrial ICC was 0.89.

### Standing long jump

The starting position of the SLJ required participants to stand with their feet shoulder-width apart behind a starting line and their arms loosely hanging down. On the command ready, set, go, participants executed a countermovement with their legs and arms and jumped at maximal effort in the horizontal direction. Participants had to land with both feet at the same time and were not allowed to fall forward or backward. The horizontal distance between the starting line and the heel of the rear foot was measured using a tape measure to the nearest 1 cm. A 90-second rest interval was provided between trials. The between-trial ICC was 0.93.

### 20-cm drop jump height and reactive strength index

Participants were instructed to place their hands on their hips and step off the platform with the leading leg straight to avoid any initial upward propulsion, ensuring a drop height of 20 cm. They were also instructed to jump with short ground contact and as forcefully as possible. Moreover, participants were instructed to leave the platform with knees and ankles fully extended and to land in a similarly extended position to ensure validity of the test. A rest period of 90 seconds was allowed between trials. The ICC for between-trial reliability was 0.95.

The RSI is a measure used in sports and fitness to assess an athlete’s ability to rapidly change from an eccentric phase to a concentric phase of movement. It’s particularly relevant in activities requiring quick changes in direction or high-intensity actions, such as tennis. RSI was calculated as follows: RSI = jump height (cm)/ground contact time (s)

### Repeated sprint ability

The RSA test was assessed via the same photocell system used for the linear speed and 505 COD speed tests (wittygate, Microgate, SRL, Bolzano, Italy). Immediately after a standardised warm-up, participants completed a preliminary single shuttle-sprint test (15+15 m with 180° COD). The first trial provided the criterion score for the actual shuttle-sprint test [18]. Participants then rested for five minutes before starting the RSA test. During the first sprint, participants had to achieve at least 97.5% of their criterion score, otherwise, they rested for five minutes and then restarted the test [18]. We used such an approach to determine if participants adopted a coping strategy during the performance of the test. Of note, all participants attained their criterion score during the first sprint. All performed six 15-m shuttle sprints with 180º turns, separated by 14 seconds of passive recovery [18]. Three seconds prior to the commencement of each sprint, players were asked to adopt the ready position using a split stance, with their front foot 0.3 m behind the starting line, until the next start signal. From the starting line, they sprinted for 15 m and touched the second line with one foot before performing a 180° COD and returning to the starting line as quickly as possible. Participants were instructed to complete all sprints as fast as possible. The RSA_best_, RSA_mean_ and RSA_total_ time were determined. Due to the fatigue induced by the test, only one maximal attempt was made i.e., no ICC was calculated.

### Statistical analyses

Data are presented as means and standard deviations (SD). The normality assumption was tested using the Shapiro-Wilk test. To establish the effect of the interventions on the dependent variables, a 2 (group: HSDT and CG) × 2 (time: pre, post) ANOVA with repeated measures was conducted for each parameter. When group × time interactions reached the level of significance (i.e., significant F value), group-specific post-hoc tests (i.e., paired t-tests) were used. The alpha level of significance was set at p < 0.05. Effect sizes (ES) were calculated from partial eta-squared values and classified according to Hopkins et al. [19]. Between-trial reliability was assessed using the ICC. The SWC was calculated as 0.2 * SD pooled, where SD represents the pooled standard deviation of pretraining scores. All data analyses were performed using SPSS 25.0 (SPSS, Inc, Chicago, IL, USA).

## RESULTS

All participants completed the study as allocated, with no training- or test-related injuries reported. At baseline, no significant betweengroup differences were observed in anthropometric characteristics, maturity offset, or physical fitness measures, and all participants were at the pubertal stage (Tables 1 and 3). Significant interactions were found for the 20-m sprint (ES = 1.17 [large], p < 0.05), 505 COD speed test (ES = 1.19 [moderate], p < 0.05), agility (ES = 2.14 [large], p < 0.01), CMJ (ES = 1.42 [large], p < 0.05), SLJ (ES = 1.05 [moderate], p < 0.05), and RSI (ES = 1.71 [large], p < 0.01), while the DJ-20 showed a tendency toward significance (ES = 0.87 [moderate]; p = 0.05). Post-hoc analyses revealed substantial improvements in the HSDT group across all significant measures: 20-m sprint (∆3.81%; p < 0.001; ES = 2.03), 505 COD speed (∆–6.02%; p < 0.01; ES = 1.62), agility (∆–6.28%; p < 0.01; ES = 1.14), CMJ (∆8.00%; p < 0.01; ES = 1.29), SLJ (∆7.80%; p < 0.01; ES = 1.13), DJ-20 (∆10.84%; p < 0.05; ES = 0.61), and RSI (∆20.07%; p < 0.01; ES = 0.99). No significant changes were observed in the control group for any measure, except for a significant decline in RSI (∆–11.28%; ES = 1.07). Analysis indicated that 92% of HSDT participants improved beyond the SWC in the 20-m sprint (n = 13), 100% in the 505 COD speed test (n = 14), 92% in agility (n = 13), 78% in CMJ (n = 11), 78% in SLJ (n = 11), 71% in DJ-20 (n = 10), and 85% in RSI (n = 12), whereas the control group showed minimal improvements (0–33%). For repeated sprint ability (RSA), analysis indicated no significant interactions (ES = 0.09–0.44; all p > 0.05). However, all HSDT participants improved RSA_best_ beyond the smallest worthwhile change (SWC), while 72% showed improvements in RSA_mean_ and RSA_total_. In contrast, 44%, 55%, and 44% of control group participants exceeded the SWC threshold for RSA_best_, RSA_mean_, and RSA_total_, respectively. Overall, these findings suggest that HSDT elicited meaningful improvements in sprint, change-of-direction, agility, jumping, and reactive strength performance, whereas the control group demonstrated minimal changes.

**TABLE 3:** Group-specific changes in measures of physical fitness from pre-to-post.

	HSDT (n=14)				CG (n=9)				ANOVA	

	Pretest		Posttest		Pretest		Posttest		*p*-value (ES)	

	M	SD	M	SD	M	SD	M	SD	Time	Group × Time
20-m sprint (s)	3.81	0.19	3.66	0.21	3.78	0.28	3.78	0.24	< 0.05 (1.19)	< 0.05 (1.17)
505-COD speed test (s)	2.93	0.13	2.75	0.12	2.98	0.15	2.95	0.12	< 0.01 (1.71)	< 0.05 (1.19)
Y-shaped test (s)	3.27	0.22	3.07	0.24	3.14	0.17	3.27	0.19	> 0.05 (0.44)	< 0.01 (2.14)
CMJ (cm)	24.47	3.87	26.43	3.25	23.27	5.18	23.08	5.38	< 0.05 (1.17)	< 0.05 (1.42)
SLJ (m)	1.64	0.18	1.77	0.18	1.65	0.33	1.67	0.34	< 0.01 (1.25)	< 0.05 (1.05)
DJ-20 (cm)	22.79	4.94	25.26	4.30	21.73	6.13	21.08	6.14	> 0.05 (0.51)	< 0.05 (0.87)
RSI (mm/ms)	0.74	0.15	0.89	1.07	0.95	0.34	0.84	0.39	> 0.05 (0.60)	< 0.01 (1.71)
RSA_best_ (s)	6.83	0.29	6.64	0.44	6.99	0.30	6.79	0.37	< 0.05 (1.47)	> 0.05 (0.09)
RSA_mean_ (s)	7.30	0.33	7.09	0.42	7.49	0.40	7.22	0.59	< 0.05 (1.45)	> 0.05 (0.17)
RSA_total_ (s)	42.22	1.85	41.15	2.57	43.25	1.89	41.32	3.50	< 0.05 (1.57)	> 0.05 (0.44)

M: mean; SD: standard deviation; HSDT: Horizontal sprint deceleration training group; CG: control group; ES: Effect size; RSI: reactive strength index; SLJ: standing long jump; CMJ: countermovement jump; COD: change of direction; RSA_best_: Repeated sprint ability best time; RSA_mean_: Repeated sprint ability mean time; RSA_total_: Repeated sprint ability total time.

## DISCUSSION

To the best of our knowledge, this is the first study to assess the effects of a 6-week HSDT program on youth tennis players’ measures of physical fitness, linear sprint speed, change in direction, agility, jumping performance, reactive strength, and repeated-sprint ability. This study demonstrated that a short-term HSDT intervention effectively enhanced multiple physical fitness attributes in youth tennis players. Conversely, regular tennis training alone did not result in significant changes across all measures of physical fitness, except for the RSA test.

A high level of linear sprint speed over short distances is an important physical fitness attribute in tennis players [20, 21]. The findings of this study showed a large improvement (∆3.81%; ES = 2.03; p < 0.001) in 20-m sprint speed performance in youth tennis players following HSDT training, while in the active CG, no significant change was detected. Indeed, 92% (n = 13) of the HSDT group improved 20-m sprint speed performance to a level that exceeded the SWC compared to only 33% (n = 3) in the active CG. This underlines the effectiveness of HSDT in improving sprint speed performance and also shows that regular tennis training is apparently not sufficient to promote the same physical fitness component in youth tennis players. These findings are in line with those established by Moran et al. [22], who found a very large improvement in 10-m sprint speed performance after an 8-week period of either one (ES = 3.10) or two NHE sessions (ES = 2.59) in youth male soccer players. Additionally, Chaabene et al. [23] reported moderate improvements in 5-, 10-, and 20-m sprint speed performance (ES = 0.82; 0.78; 0.68, respectively) after an 8-week period of NHE training in postpubertal female handball players. These gains may be attributed to improved eccentric strength of the knee extensors and potentially increased muscle fascicle length, though this remains speculative [24, 25]. However, this is mere speculation and future studies should address the mechanisms of HSDT-related sprint speed enhancement in youth.

Both COD speed and particularly agility are distinctive features of performance in tennis [26]. Of note, modern tennis imposes high demands on COD speed and agility [1, 2]. Our results indicated a large improvement (ES = 1.62) in 505 COD performance following HSDT. In addition, individual analysis demonstrated COD performance improvements > SWC in all HSDT participants. However, none of the CG participants showed an increase that exceeded the SWC. Likewise, large agility gains were observed after the HSDT (∆–6.28%; ES = 1.14; p < 0.01) while the active CG displayed a large decline in agility (∆4.31%; ES = 1.1; p < 0.05). The results of the individual changes indicated that 92% of the HSDT group (n = 13) improved their agility performance to a level that was greater than the SWC, whereas none of the active CG participants showed improvement that exceeded the SWC. The current findings are consistent with existing literature on this topic. For example, an 8-week program of NHE integrated into regular handball training compared with regular handball training only generated a large (ES = 1.38) improvement in COD speed performance in youth female handball players [23]. In addition, Moran et al. [22] reported a large COD speed performance improvement (ES = 1.04) after 8 weeks of NHE training intervention compared to standardized soccer training in youth male soccer players. Furthermore, in recently published research, Negra et al. [14] revealed large enhancements (Δ9.90%; d = 1.72; p < 0.01) in the 505 COD speed performance following 8 weeks of HSDT in male youth handball players. The same authors indicated that 94% of the HSDT group improved 505 COD speed performance to a level that was greater than the SWC, which is similar to this study’s finding. Regarding agility, our results align with the previous literature. For example, Fiorilli et al. [27] revealed an improvement in agility performance (Y-agility test) ηp2 = 0.517) after a 6-week of isoinertial eccentric-overload training program using a flywheel inertial device in junior soccer players. Although this wasn’t directly assessed, it seems plausible to argue that HSDT resulted in increased strength of knee extensors. There is evidence that eccentric strength of knee extensors plays a key role in COD speed performance, more specifically during the deceleration phase [11, 28, 29]. Therefore, the observed gains in agility and COD speed likely result from improved eccentric braking capacity, which is fundamental for rapid changes in movement direction [30].

There is now a widespread consensus that tennis demands a high level of jumping capabilities to enable players to successfully cope with the elevated physical demands of a tennis match (serve power, reaching high ball, smash and volley, and retrieving lobs and overhead shots) [20, 21]. There is evidence that jumping performance is a valid indicator of talent identification, which can distinguish between elite and non-elite youth tennis players [20, 21]. Findings of the current study indicated moderate-to-large improvements in both jumping height and distance (d = 1.29, 1.13, and 0.61 for the CMJ, the SLJ, and the DJ-20, respectively) following HSDT. In contrast, regular tennis training did not result in any significant effects. Furthermore, individual change analysis showed that 78% of the HSDT group (n = 11) improved their vertical and horizontal jumping height to a level that exceeded the SWC compared to only 22% (n = 2) (CMJ height) and 33% (n = 3) (SLJ distance) in the active CG. For DJ-20, 71% of the HSDT (n = 10) group improved their performance to a level that exceeded the SWC compared to only 33% (n = 3) in the active CG. Consistent with our findings, Chaabene et al. [23] reported an increased CMJ height performance (∆17%; ES = 0.85) after an 8-week of NHE training in postpubertal female handball players. Likewise, Moran et al. [22] revealed a moderate (ES = 1.04) improvement in SLJ performance after an 8-week of NHE training in youth male soccer players. Recently, Negra et al. [14] examined the effects of an 8-week intervention of HSDT and revealed a large improvement (ES = 1.70) in the CMJ height performance in youth male handball players. Furthermore, a systematic review with meta-analysis by Maroto-Izquierdo et al. [31] reported significant differences in training-induced vertical jumping adaptations favouring eccentric overload flywheel resistance training versus the control condition (ES = 0.46) in healthy sports science undergraduate male students. Although no mechanistic measures were conducted in this study, it is likely that the enhancements of the jumping performance are due to increased muscle strength of knee extensors following HSDT. The marked improvements in jumping height and distance further suggest neuromuscular adaptations associated with repeated deceleration stimuli [32, 33]. Given the speculative nature of the preceding statements, further studies are needed to address the mechanisms by which HSDT produces better vertical and horizontal jumping performance. Overall, the findings of this study indicate that, in addition to NHE and flywheel resistance training, HSDT is another effective eccentric exercise modality for enhancing jumping performance in youth athletes.

It is noteworthy that the RSI mirrors the ability of an individual to produce maximal strength within a minimal timeframe [34], which is an essential physical fitness attribute in tennis. The HSDT group displayed a large (∆20.07%; ES = 1.00; p < 0.01) an improvement in RSI scorecompared to a large decrease (∆-11.28%; ES = 1.07) in the active CG. Individual analysis indicated that 85% of the HSDT group (n = 12) improved their RSI score to a level that exceeded the SWC. However, all the athletes in the CG failed to reach the level of the SWC. The RSI improvement could mainly be attributed to the eccentric strength development, which is crucial for improving RSI [35, 36]. Eccentric strength allows athletes to efficiently absorb and dissipate forces during deceleration, which is then translated into rapid concentric movements. This ability is crucial for tennis players to cope with the rapid actions required by modern tennis.

One of the main performance determinants in tennis is the ability to repeat high-speed actions (i.e., RSA) [1]. This study is the first to report the effect of HSDT on RSA in youth tennis players. The findings pointed toward similar pre-to-post improvements in RSA outcomes in both the HSDT and control groups (no significant group × time interaction). The analysis of the individual changes indicates, however, that a larger percentage of participants from the HSDT achieved gains above the SWC. More specifically, all participants of the HSDT group improved the RSA_best_ and 72% improved RSA_mean_ and RSA_total_ times to a level exceeding the SWC. For the CG, the analysis revealed that only 55%, 44%, and 55% of participants improved RSA_best_, RSA_mean_ and RSA_total_ to the level that exceeds the SWC, respectively. This indicates that while regular tennis training improved RSA performance, HSDT had a greater impact. The recent study by Negra et al. [13] in which the authors examined the effects of an 8-week HSDT program in male youth handball players reported large improvements (ES = 1.01 to 1.36) in all RSA parameters (i.e., RSA_best_, RSA_mean_, and RSA_total_) after training, with no significant changes observed in the active CG. Additionally, Chaabene et al. (10) investigated the effect of NHE training compared with an active CG on RSA outcomes (i.e., RSA_best_ and RSA_total_) in youth female handball players. They reported small improvements for RSA_total_ and RSA_best_ in the NHE training group only. Furthermore, Ishoi et al. [36] reported improvements in RSA_total_ (Δ2%), and RSA_best_ (Δ2.6%) after 10 weeks of NHE training in male soccer players. Although group differences were not statistically significant, a higher proportion of athletes in the HSDT group demonstrated meaningful improvements compared with controls, suggesting a practical advantage. The potential mechanisms underlying improvements in sprint speed and COD speed may also apply here, as both components are present in the RSA protocol used (six 15-m shuttle sprints with 180º turns). Collectively, these findings reinforce the efficacy of HSDT as a timeefficient, sport-specific method to enhance multiple fitness qualities relevant to tennis [37, 38].

### Limitations

This study is not without limitations that the reader should be aware of. The main limitation of this study is the absence of randomization, as participants were allocated by team, potentially introducing selection bias. This is due to the fact that the participants belong to two different clubs, therefore randomization was not possible. This could have led to an increased risk of bias. Therefore, replication of the current methods would be required in a future randomized controlled trial. Additionally, eccentric knee extensor strength was not directly assessed, preventing confirmation of the physiological mechanisms underlying performance changes. This is due to the lack of time and available facilities at the time of the intervention. Third, the short duration of the training intervention can be a limitation. In fact, short-duration interventions might not capture the long-term benefits or potential drawbacks of the training. Another limitation concerns the lack of systematic monitoring of both internal and external training loads. Although tennis training was standardized, it is still possible that factors other than the eccentric training influenced the observed outcomes. Future studies should include comprehensive training load monitoring to better isolate the effects of the intervention. Furthermore, future research should investigate how this training mode might affect other aspects of tennis players’ fitness, particularly aerobic capacity, to provide a more holistic understanding of its benefits. Future studies employing randomized controlled designs and including direct muscle function assessments are recommended. Finally, the findings of this study should be interpreted with caution due to the limited generalizability associated with the small, nonrandomized sample.

### Practical applications

Incorporating brief HSDT sessions (approximately 25 minutes) twice weekly during the in-season period can meaningfully enhance sprint speed, jumping, agility, and reactive strength in youth tennis players. Notably, the HSDT intervention is time-efficient and can be seamlessly integrated into regular tennis training routines with minimal disruption or additional resources. This allows coaches to target and improve key physical qualities without increasing overall training volume. In contrast, standard tennis training alone appears insufficient to elicit substantial improvements across most fitness measures, except for RSA performance, underscoring the added value of implementing short, focused HSDT sessions.

## CONCLUSIONS

In summary, six weeks of HSDT, performed twice weekly, significantly improved several key physical fitness attributes in youth tennis players, including sprint speed, agility, jumping, and reactive strength, while RSA showed no additional benefit compared to regular tennis training. HSDT seems to be an effective, practical, and time-efficient eccentric-oriented method to complement traditional tennis training. Future research should explore its long-term effects and underlying neuromuscular adaptations, considering the potential influence of maturation.

## Data Availability

Data are available from the first author upon reasonable request.
